# Prevalence of Angiogenesis, Proliferation, and Apoptosis Markers of Cervical Cancer and Their Correlation with Clinicopathological Parameters

**DOI:** 10.1155/2020/8541415

**Published:** 2020-11-12

**Authors:** Anita Ramesh, R. Vimal Chander, Chitra Srinivasan, Srinivasan Vengadassalapathy

**Affiliations:** ^1^Department of Medical Oncology, Saveetha Medical College and Hospital, Thandalam, Chennai 602105, India; ^2^Department of Pathology, Saveetha Medical College and Hospital, Thandalam, Chennai 602105, India; ^3^Department of Pharmacology, Saveetha Medical College and Hospital, Thandalam, Chennai 602105, India

## Abstract

The aim of the study is to investigate the expression of angiogenesis (VEGF and PDGF), angiogenesis inhibitor markers (angiostatin and endostatin), proliferation (Ki67), and apoptosis markers (p53 and p16) of cervical cancer in Indian population and to correlate them with the clinicopathological profile. It is a descriptive study of consecutive cases of cervical cancer from Saveetha Medical College and Hospital between January 2017 and December 2018. The expression of angiogenesis, angiogenesis inhibitor markers, Ki67, p53, and p16 in 60 cases of cervical sections were detected by the immunohistochemical method and analyzed with clinicopathological data. VEGF expression was positive in 16 cases (26.67%) and negative in 20 cases (33.33%). As of PDGF, 3 cases (3.33%) have shown positivity to PDGF and 33 cases have shown negativity. Angiostatin and endostatin expression was reported to be positive in 10 (16.67%) and 21 (35%) cases, respectively. Most of the cases 57 (95%) have shown both p16 and Ki67 positivity. Although p53 expression was positive in 48 cases (80%), the remaining 12 cases (20%) were p53-negative. The PDGF expression was significantly correlated to the stage of tumors. No statistically significant association was observed between angiogenesis inhibitor markers and clinicopathological parameters. A significant positive correlation was noticed between the Ki67 expression and stage of tumors.

## 1. Introduction

According to the WHO, cervical cancer is the fourth most common cancer among malignant tumors in women worldwide [1]. Currently, cancer is the second leading cause of death, after heart disease, and this imposes a huge burden on societies [2]. It is estimated that about 0.6 million cases and 0.3 million deaths were reported every year worldwide. Approximately 90% of deaths from cervical cancer occurred in low- and middle-income countries [3, 4]. In India, 122844 women are diagnosed with cervical cancer annually and 67477 die from the disease [5]. Human papilloma virus (HPV) infection has been determined as the main risk factor for cervical cancer. Previous clinical evidences showed that the development of cervical cancer is a multifactorial process in which HPV infection takes a central place along with other risk factors such as smoking, immunosuppression, immunodeficiency, diet, parity, age at first full term pregnancy, and family history [6].

Angiogenesis plays a key role in tumor growth and metastasis. Expression of angiogenic factors has been suggested as a marker for tumor malignancy, and it may help to identify those patients with a poorer prognosis, aiding patient stratification for more aggressive and/or angiogenesis-targeted therapy. Carcinogenesis is a complex and multistep process accompanied by multiple genetic changes including all oncogenes, tumor suppressor genes, and growth factor genes [7]. Estrogen receptor (ER) and progesterone receptor (PR) are present on the surface of hormone target cells and are mainly distributed in targets such as uterus and cervix. These can specifically bind to corresponding hormones and play a role in regulation of occurrence and development of cervical cancer [8]. Ki67 is a nuclear protein that is associated with cell proliferation and has been suggested as a sensitive biological indicator of cancer progression. The p16INK4a protein (p16) is a cyclin-dependent kinase inhibitor, which negatively regulates progression through the G1-S transition checkpoint of the cell cycle [9]. The p53 gene is an important tumor suppressor gene and plays a major role in cell division and differentiation [10].

The expression of these biomarkers is associated with the severity and progression of cervical cancer. The use of molecular markers has aided histopathology to identify women at high risk of recurrence and in the definition of doubtful cases. Furthermore, cancer development, metastasis, and progression are mainly dependent on vasculogenesis and angiogenesis, and we hypothesize that markers of tumor angiogenesis in the early stage of carcinogenesis not only influenced the biology of the disease but also increased the response to treatment and patient outcome. The purpose of the present study was to determine the prevalence of angiogenesis, angiogenesis inhibitor, proliferation, and apoptosis markers in Indian population and to correlate them with clinicopathological parameters (age, stage, grade, and histopathological diagnosis).

## 2. Materials and Methods

### 2.1. Study Population

Consecutive cases of the Saveetha Medical College and Hospital, Thandalam, Chennai, from January 2017 to December 2018, were reviewed descriptively, and the information of cervical cancer cases was abstracted using a standard form. Biopsy samples were sent to the Department of Pathology for histopathological examination for diagnosis and pathologic staging. Macroscopic and microscopic features of the specimens were noted, and a total of 60 histologically confirmed cases of cervical carcinoma during this period were included for this study. Cases without clear pathological examination were excluded. Moreover, patients without written consent were excluded.

### 2.2. Clinicopathological Factors

Clinicopathological factors such as age, tumor size, lymph node metastasis, histological grade, and lymphovascular invasion are the established prognostic factors for cervical cancer. Histologic grading was performed morphologically into well-differentiated, moderately differentiated, and poorly differentiated tumors, depending on the degree of resemblance of the tumor cells to the mature squamous cells. They were then staged according to the FIGO staging system [11].

### 2.3. Histological Evaluation

#### 2.3.1. Immunohistochemistry Staining Protocol

1. Cut and mount 3-4 micron formalin-fixed paraffin-embedded tissues on positive-charged slides
2. Air dry for 2 hours at 58 degree Celsius
3. Deparaffinise, dehydrate, and rehydrate the tissues
4. Subject tissues to heat epitope antigen retrieval using a suitable retrieval solution with citrate or EDTA (ethylenediamine tetra acetic acid)
5. Wash with 5 changes of IHC (immunohistochemistry) wash buffer
6. Place slides in PolyDetector Peroxidase Blocker for 5 min
7. Wash with 3 changes of IHC wash buffer
8. Cover tissue with primary antibody following manufacturer's recommended protocol. If using concentrated antibodies, use ImmunoDetector Protein Blocker/Antibody Diluent to dilute antibodies.
9. Wash with 3 changes of IHC wash buffer
10. Cover tissue with PolyDetector HRP (horseradish peroxidase) label and incubate for 45 min
11. Rinse with 3 changes of IHC wash buffer
12. Prepare DAB (diaminobenzidine) by adding one drop of PolyDetector DAB Chromogen per ml of PolyDetector DAB Buffer and mix
13. Cover the tissue with prepared DAB substrate-chromogen solution and incubate for 10 min
14. Rinse with 5 changes of distilled water
15. Counterstain and then dehydrate
16. Coverslip

### 2.4. Immunohistochemical Evaluation

Immunohistochemical analysis (IHC) for angiogenesis markers such as the vascular endothelial growth factor (VEGF) and platelet-derived growth factors (PDGF), angiogenesis inhibitor markers (endostatin and angiostatin), proliferation marker (Ki67), apoptotic marker (p53), and p16 were performed on representative sections from all cases. The HRP polymer detection kit from Leica Microsystems was used in this study to detect the bound antibody with diaminobenzidine as the chromogen. Slides were counterstained with Harris hematoxylin, and the results were evaluated with positive and negative tissue controls. The immunohistochemical expression was scored using the Allred scoring system, incorporating the proportion and intensity scores. High and extremely high expression of Ki67 proliferative index was defined as nuclear expression ≥10% and ≥30% of the tumor cells, respectively. Assessment of mutant p53 status is performed using the IHC Allred scoring system taking into consideration the staining intensity and percentage of tumor cells showing nuclear positivity as follows.

#### 2.4.1. Allred Scoring System

(i) Intensity score
  1. Score 1: mild
  2. Score 2: moderate
  3. Score 3: strong
(ii) Proportion score (percentage of tumor cells showing nuclear positivity)
  1. Score 1: <1%
  2. Score 2: 1–10%
  3. Score 3: 11–33%
  4. Score 4: 34–66%
  5. Score 5: 67–100%
(iii) Overall score = intensity score + proportion score
(iv) Usually staining of >5% of nuclei is considered positive [12]

### 2.5. Statistical Analysis

Statistical analysis was performed by estimating the prevalence of angiogenesis, angiogenesis inhibitor, proliferation, and apoptosis markers and correlating with clinicopathological parameters. All the statistical analyses were performed using the software STATA. Descriptive statistics such as percentages and frequencies were calculated. Relationship between two variables was performed using the Pearson correlation test. The correlation coefficient value ranges from −1 to +1, the negative value represents that the parameters are inversely proportional to each other, and the positive value represents that the parameters are directly proportional to each other. The extent to which this correlation coefficient can show a significance was given in terms of *p* value. *p* value of <0.05 was taken as statistically significant, and the data were represented in the form of tables.

## 3. Results

From January 2017 to December 2018, a total of 60 cases of cervical cancer were included and analyzed. All studied cases were of age between 37 and 74 years. Mean age of the population at the time diagnosis was 53.93 ± 10.24 years. In our study, most reported histological type was squamous cell carcinoma (52/60, 86.67%) followed by adenocarcinoma (5/60, 8.33%) and one case each of carcinosarcoma, microinvasive squamous cell carcinoma, and squamous cell carcinoma, large cell nonkeratinizing type.  Table 1 describes the clinicopathologic characteristics of patients.

For histological grading of cervical cancer, Nottingham's histologic scoring system was used. Out of total 60 cases, the highest number of cases (44) was presented with histological grade II (73.33%), followed by grade I (13.33%) and grade III (13.33%). Based on the tumor staging system, most of the patients were characterized as stage II (44/60, 73.33%) and stage I (9/60, 15%).

In present study, expression of angiogenesis (VEGF and PDGF), angiogenesis inhibitor markers (angiostatin and endostatin), proliferation marker (Ki67), and apoptosis markers (p53 and p16) was detected using immunohistochemical analysis. Out of total 60 observed cases of cervical cancer, expression of angiogenesis and angiogenesis inhibitor markers was noticed in 36 cases, and the remaining cases were excluded due to background staining of the antibody. Among 36 cases, positive expression of VEGF was observed in 16 cases (26.67%) and 20 cases showed negativity to VEGF. In the identification of the PDGF factor, 3 cases (3.33%) have shown positivity to PDGF, and 33 cases have shown negativity.

Table 2 presents the summary statistics of cancer-related markers. In a total of 36 cases, angiostatin and endostatin expression were reported as positive in 10 (16.67%) and 21 (35%) cases, respectively. Out of total 60 observed cases, 48 cases (80%) showed p53 positivity, and 12 cases (20%) showed negativity to p53. As shown in Table 2, about 57 cases (95%) have shown p16 positivity and remaining 5% shown negativity, which included cases of MMMTs carcinosarcoma (malignant-mixed Mullerian tumor) and well-differentiated adenocarcinoma, probably endocervical adenocarcinoma. As regards to Ki67, 95% cases have shown positivity.

During the study period, we estimated the correlation between various cancer-related markers and clinicopathological parameters. As shown in Table 3, most of the parameters showed no significant correlation with angiogenesis markers. The stage was statistically associated with the PDGF marker (*p* = 0.0112, 0.41797) and showing that the stage is positively associated with the expression of PDGF.

Table 4 shows the correlation coefficients of angiogenesis inhibitor markers with various clinicopathological parameters. No statistically significant correlation (*p* < 0.05) was noticed between angiogenesis inhibitor markers (angiostatin and endostatin) and the clinicopathological parameters such as age, stage, and grade. As shown in Table 5, a statistically significant correlation was seen between the proliferation marker (Ki67) and stage (*p* = 0.0030, 0.37697), showing that the expression of Ki67 was positively correlated with stage of tumors. However, we did not find any correlation between apoptosis markers (p53 and p16) and age, stage and histological grade as observed in Table 6.

## 4. Discussion

Cervical cancer is one of the most dreadful conditions in the world of gynae-oncology. Development of cervical cancer is a complex process that develops from normal to inflammatory to tumors, accompanied by multiple genetic changes. Several clinical studies have shown that VEGF expression and angiogenesis play a prognostic role in advanced squamous cell carcinoma, being associated in most cases with poor prognosis and decreased survival [13–16]. The present study was designed to estimate the prevalence of angiogenesis, proliferation, and apoptosis markers in Indian population and to correlate them with clinicopathological parameters.

Cervical carcinoma can occur in of all ages of females; however, its usual age at presentation is 35–55 years with the peak age for the incidence varying with populations [17]. In current study, the mean age was 53.93 ± 10.24 years at diagnosis ranging from 37 to 74 years. Most of studies have observed maximum cases in elder women >40 years of age [18, 19]. The most common age group involved in cervix carcinoma ranged from 35 to 50 years [20]. One study reported that incidence rises in 30–34 years of age and peaks at 55–65 years [21].

In the present study, most of the patients (44, 73.33%) presented with grade II followed by grade I and III. Majority of patients were defined as stage II (44/60, 73.33%) and stage I (9/60, 15%). In agreement with our study findings, a study by Goellner et al. [22] suggested that the most common presenting staging for cervical carcinoma is stage 1, and 74.5% of the patients have grade 3 disease.

Majority of cervical cancers were squamous cell carcinomas. These lesions arise from the squamocolumnar junction and may be keratinizing or nonkeratinizing type (well-differentiated to poorly differentiated carcinoma). Studies have shown that 85–90% of cases of cervical carcinoma are squamous cell carcinoma and rest of them constitutes adenocarcinoma [18, 23]. Adenocarcinoma of the uterine cervix arises from the endocervical columnar cells and account for about 14% of cervical carcinomas [24].

Out of total 36 cases, 16 cases (26.67%) were observed as a positive to VEGF and 20 cases (33.33%) showed negativity. It is similar to a study conducted by Kfouri et al. [25] who noticed that 16 patients were positive for VEGF protein and 24 patients were considered negative due to low expression. However, other study reported higher rates of VEGF expression (66%), used previous published histoscore ≥200 for defining positivity of VEGF expression compared to our study [26]. VEGF production is considered to be essential for angiogenesis and cancer metastasis. Early assessment of VEGF expression provides additional information for recognition of cervical cancer patients who had a low likelihood of response to neoadjuvant chemotherapy and an unfavorable prognosis [27]. With regards to PDGF, 3 cases (3.33%) were positive for PDGF and 33 cases were shown negative to PDGF, which is in consistent to the study performed by Taja-Chayeb et al. [28] on expression of PDGF in cervical cancer. As regards to angiogenesis inhibitor markers, angiostatin and endostatin expression was positive in 10 (16.67%) and 21 (35%) cases, respectively. Out of total 60 observed cases, 80% have shown positivity to p53 and 20% have shown negativity. In our study, 95% of cases show both p16 and Ki67 positivity. In similar to our study findings, a study conducted by Kanthiya et al. [29] demonstrated p16 and Ki67 expressions in 85 cases (35.0%) and 99 cases (40.7%), respectively. The expression of p16 and Ki67 was higher (87%) in cervical intraepithelial neoplasia (CIN 3). The variation of expression rates may partly depend on the criteria defining positive expression. A systematic review on immunohistochemical expression of p16, Ki67, and p53 in cervical lesions reported that nineteen out of 22 evaluated studies have shown that there is a higher p16 expression in more severe cancer lesions, while in p53 expression, only 4 out of the 9 studies showed a higher expression among more severe cases. Regarding the Ki67 expression, it was observed that 9 out of 14 studies showed higher expression in more severe lesions [10]. Based on these studies, it is confirmed that rates of p16 and Ki67 expressions were directly associated with the severity of cervical lesions.

Over the 2-year study period, we evaluated the correlation between cancer-related markers and clinicopathological factors. In our study, most of the parameters showed no statistically significant correlation with angiogenesis markers (VEGF and PDGF). Although PDGF was correlated significantly with stage (*p* = 0.0112, 0.41797). In contrast to our findings, a study by Goncharuk et al. [30] reported a statistically significant correlation between the high level of VEGF expression and lymph node status (*r* = 0.39, *p* < 0.05).

In present study, no statistically significant correlation (*p* < 0.05) was noticed between angiogenesis inhibitor markers (angiostatin and endostatin) and the clinicopathological parameters such as age, stage, and grade. There was a significant correlation between the Ki67 expression and stage (*p* = 0.0030, 0.37697), which is consistent to the study performed by Amaro-Filho et al. [31] who observed significant increase trend of Ki67 expression with an increased FIGO stage (nptrend = 0.008).

## 5. Conclusion

In conclusion, the present study revealed several correlations between cancer-related markers and clinicopathological parameters. No statistically significant correlation was noticed between angiogenesis markers and clinicopathological factors. Although PDGF was correlated significantly with stage of tumors, angiogenesis inhibitor markers were not significantly correlated to age, stage, and grade.

A significant correlation was noticed in this study between the Ki67 expression and stage of tumors. However, further investigation is required to determine the prevalence of angiogenesis inhibitor markers, apoptosis, and proliferation markers and its correlation with the clinicopathological profile of cervical cancer in Indian population.

## Figures and Tables

**Table 1:** Summary of clinicopathologic characteristics of patients.

Parameter	Value

Age (years)	
*N*	60
Mean	53.93
Standard deviation	10.24
Minimum	37.00
Maximum	74.00

Sex, *N* (%)	
*F*	60 (100.00%)

HPE diagnosis, *N* (%)	
Adenocarcinoma	5 (8.33%)
Carcinosarcoma (malignant-mixed Mullerian tumor)	1 (1.67%)
Microinvasive squamous cell carcinoma	1 (1.67%)
Squamous cell carcinoma	52 (86.67%)
Squamous cell carcinoma, large cell nonkeratinizing type	1 (1.67%)

Histologic grade, *N* (%)	
I	8 (13.33%)
II	44 (73.33%)
III	8 (13.33%)

Stage, *N* (%)	
I A	2 (3.33%)
I B	7 (11.67%)
II A	6 (10.00%)
II B	38 (63.33%)
III A	1 (1.67%)
III B	4 (6.67%)
IV A	2 (3.33%)

**Table 2:** Summary statistics of cancer-related markers.

Biomarker intensity	Value *N* (%)
Angiogenesis markers	

****Vascular endothelial growth factor (VEGF)	

****Mild	2 (3.33%)

****Moderate	4 (6.67%)

****Negative	20 (33.33%)

****Strong	10 (16.67%)

****Platelet-derived growth factor (PDGF)	

****Moderate	1 (1.67%)

****Negative	33 (55.00%)

****Strong	2 (3.33%)

Angiogenesis inhibitor markers	

****Angiostatin	

****Mild	6 (10.00%)

****Moderate	4 (6.67%)

****Negative	26 (72.22%)

****Endostatin	

****Mild	7 (11.67%)

****Moderate	9 (15.00%)

****Negative	15 (25.00%)

****Strong	5 (8.33%)

Proliferation marker	

****KI67	

****Moderate	1 (1.67%)

****Negative	3 (5.00%)

****Strong	56 (93.33%)

Apoptosis marker	

p53	

Moderate	13 (21.67%)

Negative	12 (20.00%)

Strong	35 (58.33%)

p16	

****Mild	1 (1.67%)

****Moderate	17 (28.33%)

****Negative	3 (5.00%)

****Strong	39 (65.00%)

**Table 3:** Correlation coefficients of angiogenesis markers with various factors.

Parameters	Correlation coefficient	*p* value

VEGF with age (*n* = 24)	−0.32810	0.1175
VEGF with stage (*n* = 24)	0.06884	0.7492
VEGF with histologic grade (*n* = 24)	0.05822	0.7870
PDGF with age (*n* = 36)	0.03842	0.8240
PDGF with stage (*n* = 36)	0.41797	0.0112
PDGF with histologic grade (*n* = 36)	0.02889	0.8672

**Table 4:** Correlation coefficients of angiogenesis inhibitor markers with various factors.

Parameters	Correlation coefficient	*p* value

Angiostatin with age (*n* = 36)	0.25428	0.1345
Angiostatin with stage (*n* = 36)	0.25697	0.1303
Angiostatin with histologic grade (*n* = 36)	0.18946	0.2684
Endostatin with age (*n* = 36)	−0.06229	0.7182
Endostatin with stage (*n* = 36)	0.21731	0.2030
Endostatin with histologic grade (*n* = 36)	0.04353	0.8010

**Table 5:** Correlation coefficients of proliferation markers with various factors.

Parameters	Correlation coefficient	*p* value

KI67 with age (*n* = 60)	0.16668	0.2031
KI67 with stage (*n* = 60)	0.37697	0.0030
KI67 with histologic grade (*n* = 60)	0.18394	0.1595

**Table 6:** Correlation coefficients of apoptosis markers with various factors.

Parameters	Correlation coefficient	*p* value

p53		
p53 with age (*n* = 60)	0.07114	0.5891
p53 with stage (*n* = 60)	−0.04702	0.7213
p53 with histologic grade (*n* = 60)	NA	NA
p16		
p16 with age (*n* = 60)	0.15003	0.2525
p16 with stage (*n* = 60)	−0.15921	0.2243
p16 with histologic grade (*n* = 60)	−0.13738	0.2952
NA, not applicable		

## Data Availability

The data used to support this study are available from the corresponding author upon request.
